# Mortaparib, a novel dual inhibitor of mortalin and PARP1, is a potential drug candidate for ovarian and cervical cancers

**DOI:** 10.1186/s13046-019-1500-9

**Published:** 2019-12-19

**Authors:** Jayarani F. Putri, Priyanshu Bhargava, Jaspreet Kaur Dhanjal, Tomoko Yaguchi, Durai Sundar, Sunil C. Kaul, Renu Wadhwa

**Affiliations:** 1 DBT-AIST International Laboratory for Advanced Biomedicine [DAILAB], DAICENTER, National Institute of Advanced Industrial Science and Technology [AIST], Central 5-41, Higashi 1-1-1, Tsukuba, Ibaraki 305 8565 Japan; 2 DAILAB, Department of Biochemical Engineering and Biotechnology, Indian Institute of Technology [IIT] Delhi, New Delhi, Hauz Khas 110 016 India

**Keywords:** Mortaparib, Mortalin, p53, PARP1, Inhibitor, Growth arrest

## Abstract

**Background:**

Mortalin is enriched in a large variety of cancers and has been shown to contribute to proliferation and migration of cancer cells in multiple ways. It has been shown to bind to p53 protein in cell cytoplasm and nucleus causing inactivation of its tumor suppressor activity in cancer cells. Several other activities of mortalin including mitochondrial biogenesis, ATP production, chaperoning, anti-apoptosis contribute to pro-proliferative and migration characteristics of cancer cells. Mortalin-compromised cancer cells have been shown to undergo apoptosis in in vitro and in vivo implying that it could be a potential target for cancer therapy.

**Methods:**

We implemented a screening of a chemical library for compounds with potential to abrogate cancer cell specific mortalin-p53 interactions, and identified a new compound (named it as Mortaparib) that caused nuclear enrichment of p53 and shift in mortalin from perinuclear (typical of cancer cells) to pancytoplasmic (typical of normal cells). Biochemical and molecular assays were used to demonstrate the effect of Mortaparib on mortalin, p53 and PARP1 activities.

**Results:**

Molecular homology search revealed that Mortaparib is a novel compound that showed strong cytotoxicity to ovarian, cervical and breast cancer cells. Bioinformatics analysis revealed that although Mortaparib could interact with mortalin, its binding with p53 interaction site was not stable. Instead, it caused transcriptional repression of mortalin leading to activation of p53 and growth arrest/apoptosis of cancer cells. By extensive computational and experimental analyses, we demonstrate that Mortaparib is a dual inhibitor of mortalin and PARP1. It targets mortalin, PARP1 and mortalin-PARP1 interactions leading to inactivation of PARP1 that triggers growth arrest/apoptosis signaling. Consistent with the role of mortalin and PARP1 in cancer cell migration, metastasis and angiogenesis, Mortaparib-treated cells showed inhibition of these phenotypes. In vivo tumor suppression assays showed that Mortaparib is a potent tumor suppressor small molecule and awaits clinical trials.

**Conclusion:**

These findings report (i) the discovery of Mortaparib as a first dual inhibitor of mortalin and PARP1 (both frequently enriched in cancers), (ii) its molecular mechanism of action, and (iii) in vitro and in vivo tumor suppressor activity that emphasize its potential as an anticancer drug.

## Introduction

Mortalin/GRP75/HSPA9/mthsp70 is a ubiquitously expressed member of heat shock protein 70 family of chaperones and has been established to play key roles in number of biological processes including cell proliferation, migration, angiogenesis, protein folding, chaperoning, intracellular trafficking, mitochondrial biogenesis, apoptosis and carcinogenesis [1–5]. It is overexpressed in almost all types of cancers and regulates several properties of cancer cells including anti-apoptosis, migration, invasion, metastasis and drug resistance [5–7]. Mortalin was shown to inhibit tumor suppressor p53 function by interacting with it in the cytoplasm [8] as well as in nucleus [2, 9], where it controls centrosome duplication, activates telomerase and hnRNPK yielding malignant properties of cancer cells [2]. Of note, mortalin-p53 interactions were shown to be specific to cancer cells [8, 10]. Consistently, mortalin shRNA caused apoptosis in hepatocarcinoma, but not in primary liver cells [11]. Furthermore, some potential anticancer drugs were shown to activate p53 function and also caused shift in mortalin staining pattern from perinuclear (typical of cancer cells) to pancytoplasmic (typical of normal cells) [12–14]. Gao et al. [14] showed that mortalin shRNA caused induction of DNA damage signaling, leading to growth arrest of cancer cells. Proteins such as PARP1, BRCA1, ATM, ATR, CHK1, CHK2, RAD9 and p53 regulate DNA damage response for the maintenance of genome integrity, and are often mutated or dysregulated in large variety of cancers [15]. Enforcement of DNA damage response by either the reconstitution of mutated DNA damage regulatory proteins or inhibition of repair signaling has been shown to cause growth arrest/apoptosis of cancer cells, and hence considered as a potential strategy for cancer therapy [16]. Poly ADP-Ribose Polymerase (PARP)-1 is a 116-kDa nuclear protein that plays a key role in sensing DNA damage and its repair by recruitment of DNA repair machinery [17]. It has also been shown to have function in various cellular contexts including metabolism, aging, inflammation, epigenetic modification, apoptosis and metastasis [18–21]. Function of PARP1 for recruitment of DNA repair machinery has been shown to be dependent on its PARylation, addition of sugar moiety poly (ADP-ribosyl) (PAR), that requires NAD^+^ and its removal by PARG (Poly(ADP-ribose) glycohydrolase) [21]. The latter is a nuclear and mitochondrial protein that removes PAR and restores PARP1 for next round of DNA repair [22].

Olaparib, Niraparib, Rucaparib, Talazoparib and Veliparib are FDA approved drugs [23, 24] for PARP1 inhibition in BRCA1 mutated cancers. Currently, some other drug candidates, like simmiparib, are undergoing development in clinical phase trials [25] for assessing long term toxicity to many organs and resistance in clinical settings. Mechanistically, PARP1 inhibitors have been shown to compete with ligand NAD^+^, inhibit the PARylation of PARP1 or restoration by PARG enzyme resulting in increase in accumulation of DNA double-strand breaks (DSB) [26] leading to growth arrest/apoptosis in cancer cells. Components of DNA damage and oxidative signaling have been reported to mediate such growth arrest/apoptosis. These include activation of NFkB (Nuclear Factor kappa B) [27], Caspases, ATM (ataxia telangiectasia-mutated) and p53 [28, 29]. In contrast to the NAD^+^ dependent C-terminal inhibition of PARP1, DNA trapping mediated by its N-terminal has been reported as another independent way of its inactivation [30]. Olaparib, Simmiparib, Veliparib and MK4827 have been reported to cause N-terminal inhibition of PARP1 leading to growth arrest/apoptosis of cancer cells [25, 30]. Furthermore, PARP1 inhibitors have been shown to sensitize cancer cells to other anticancer drugs and hence deemed beneficial for combinatorial therapies [31, 32].

By using abrogation of mortalin-p53 interaction as a screening assay, we isolated a compound (5-[1-(4-methoxyphenyl)(1,2,3,4-tetraazol-5-yl)]-4-phenylpyrimidine-2-ylamine; named Mortaparib) that causes nuclear enrichment and activation of p53 function through deregulation of mortalin. We found that the transcriptional repression of mortalin by Mortaparib caused inactivation of PARP1 leading to accumulation of DSB (Double-Strand Breaks) and apoptosis in cervical cancer cells. We present in silico and experimental evidences (in vitro and in vivo) for Mortaparib as a first potential dual inhibitor of mortalin and PARP1 for treatment of cervical and ovarian cancers.

## Material and methods

### Cells, plasmids and transfection

Human normal cells (MRC5 and TIG3), cervical carcinoma (HeLa, ME180, SKG-II and SKG-IIIb), breast carcinoma (MCF7), osteosarcoma (U2OS), colorectal adenocarcinoma (DLD1), human lung carcinoma (A427 and A549), non-small lung cancer (NCI-H1299), ovarian cancer (SKOV3 and OVK 18–1), human prostate cancer (PC3 and DU145), hepatoma (PLC) and human pancreatic (Suit-2) cancer cell lines were bought from the Japanese Collection of Research Bioresources Cell Bank (JCRB) or DS Pharma, Japan and grown in DMEM/RPMI/MEM medium-supplemented with 10% FBS, 1% penicillin-streptomycin, and incubated in a humidified incubator maintained at 5% CO_2_ and 37 °C.

Cells stably expressing full-length mortalin protein were prepared, as previously described [33]. Mortalin-targeting shRNA (shRNA-2166) was used for knockdown of mortalin, as described previously [10, 11]. The PG13-luc (firefly luciferase reporter driven by p53-binding consensus sequence) and pWWP-luc (firefly luciferase reporter gene driven by p21^WAF1^ promoter) were used for reporter assays to investigate the transcriptional activation function of p53, as decribed previously [8]. The siRNA for silencing human PARP1 was purchased from Thermo Fisher Scientific, Inc. (Life and Technologies, Japan).

For transfections, cells were grown in 6-well culture plates and transfected with indicated plasmids at 70% density using Lipofectamine, following the manufacturer’s instruction (Invitrogen, CA, USA). After 24 h of transfection, the medium was replaced with fresh medium with/without the drug (as indicated) for 24 h. Control and transfected cells were subjected to molecular analyses.

### Library screening

A library of 12,000 compounds (synthetic and natural) was screened for candidates capable of abrogating mortalin-p53 interactions resulting in nuclear enrichment of p53 and shift in mortalin staining pattern from perinuclear (concentrated around the nuclear membrane; typical of cancer cells) to pancytoplasmic (widely distributed in the cell cytoplasm; typical of normal cells) type. Cells (MCF7 and U2OS) were seeded in 96-well plates, treated with sub-toxic doses of compounds followed by fixation with acetone:methanol (1:1) and double immunostaining for mortalin and p53 using specific antibodies. Immunostaining was recorded using the automated scanning system attached to Axiovert 200 M microscope (Zeiss). Selected compounds that caused shift in mortalin staining pattern and enrichment of nuclear p53 were screened again for 4 rounds. Finally, 6 compounds out of 12,000 were finally selected that showed similar results in two cell lines.

### Luciferase reporter assay

Cells were transfected with reporter plasmid, as indicated, followed by treatment with drugs, harvested and lysed in passive lysis buffer (Promega, Japan). Protein lysates were subjected to quantification using bicinchonic acid assay (BCA) (Thermofisher Scientific, Rockford, IL). Luciferase activity was measured using Dual-Luciferase® Reporter Assay System (Promega, Japan) following the manufacturer’s instructions. The pRL-TK vector was used as an internal control for transfection efficiency.

### Cell viability assay

Cells were seeded in 96-well culture plate and treated with Mortaparib or Olaparib for 24 h. Mortalin overexpressing and knockdown variants of MCF7 cells were seeded in 96-well culture plate and treated with Mortaparib for 24 h. Culture medium was replaced with fresh medium and MTT [3-[4, 5-Dimethylthiazol-2-yl]-2, 5-diphenyltetrazolium bromide] (5 mg/ml in PBS) was added to each well and incubated for 3 h. MTT solution was replaced with DMSO (100 μl) and chromogen was quantitated at 570 nm using spectrophotometer (Tecan, Switzerland). The experiment was repeated thrice. Statistical analysis was carried out using QuickCals t-test calculator (GraphPad Software, Inc., CA).

### Colony formation assay

Cells (5 × 10^2^ per well) were seeded in 6-well culture plates for 24 h, and treated with Mortaparib every alternate day up to 10 days. Colonies were washed with PBS, fixed with pre-chilled methanol-acetone (1:1) at 4 °C for 10 min, and stained with 0.1% Crystal violet (Sigma-Aldrich, Germany). Plates were washed, dried and photographed. The number of colonies were counted manually. The experiment was repeated at least thrice for statistical significance.

### Immunostaining

Cells were cultured on cover slips (18-mm) placed in 12-well culture plates and after 24 h of Mortaparib treatment, fixed in acetone:methanol (1:1) at 4 °C for 5 min. Fixed cells were permeabilized with 0.2% Triton X-100 in PBS (PBST) and blocked with 2% BSA in PBS. Anti-PARP1/2 (H-250), −p300 (c-20), −p53 (DOI), −Histone 3 (FL-136), −p38α (sc-535), −CDK4 (c-22), −Cyclin D1 (DSC-6), −E2F1 (KH-95), −BCL2 (N-19) (Santa cruz, CA, USA), −BRCA1/2 (Abcam 16,781), −Cytochrome c (Abcam 133,504), −COX 1 (Invitrogen 35–8100), −PAR (Abcam 14,459), −H2AX (Upstate, Millipore), p-H2AX (Cell Signaling Tech, 20E3), −Caspase 3 (BD transduction Lab.), −p21^WAF1^ (Cell Signalling Tech, D12), −COX IV (Cell Signalling Tech, 3E11), −pSAP/JNK (Cell Signaling Tech, 81E11), −cleaved PARP1 (Cell Signaling Tech, D214), and -pRb (Cell Signalling Tech, S780) antibodies were used. Anti-mortalin antibodies were raised in our laboratory. Fixed cells were incubated with indicated antibodies (1–3 μg/mL) at 4 °C overnight followed by three (10 min each at room temperature) washings with PBST and incubation with fluorochrome-conjugated secondary antibodies (Alexa-488-conjugated goat anti-rabbit, anti-mouse, Alexa-594-conjugated goat anti-rabbit or anti-mouse (Molecular Probes, OR)) for 30 mins. Nucleus was counter-stained with Hoechst 33342 (Thermo Fisher Sci). Cells in coverslips were washed three times with PBS and mounted with FA mounting fluid (VMRD Inc., Pullman, WA). Cells were observed using Zeiss Axiovert 200 M microscope (Carl Zeiss Microimaging, Thornwood, NY) and analysed by AxioVision 4.6 software (Carl Zeiss Microimaging). ImageJ (NIH, USA) software was used to quantify fluorescence intensity.

### Western blot analysis

Control and Mortaparib-treated cells were harvested and lysed with RIPA buffer (Thermo Fisher Scientific, Rockford, IL) containing protease inhibitors (Roche Applied Science, Mannheim, Germany). The protein concentration was measured using bicinchonic acid (BCA) assay (Thermo Fisher Scientific, Rockford, IL). Protein lysates (~ 20 μg) were resolved on SDS-polyacrylamide gels (MiniProtean Bio Rad, CA, USA) and transferred to PVDF membrane (Immobilon Merck, Germany) by semi-dry transfer system (Powered Blot One, ATTO, Japan). Membranes were blocked in 0.2% Tween 20 in TBS (TBST) containing 3% BSA incubated with primary antibodies (1–3 μg/mL) (as indicated) at RT for 45 min or at 4 °C overnight. Later, membranes were washed with TBST three times and incubated with horseradish peroxidase-conjugated secondary antibodies (1–3 μg/mL) (Santa Cruz, CA, USA). The protein bands were developed using ECL (GE Healthcare Life Sci., UK) and visualized using Gel Doc Documentation (Bio-Rad, CA. USA). ImageJ (NIH, USA) software was used to quantify protein signals.

### Detection of DNA double strand breaks

DNA double strand breaks were detected using neutral CometAssay Kit (Trivagen, MD, USA) following the manufacturer’s instructions. Briefly, control and Mortaparib-treated cells were harvested and mixed with pre-warmed (37 °C) agarose, and layered immediately onto Cometslide™ (Trivagen). Slides were placed in dark at 4 °C for 1 h and immersed in lysis solution at 4 °C overnight. Excess of lysis solution was removed and slides were subjected to electrophoresis using Neutral Electrophoresis Buffer™ (Trivagen). The slides were then washed with 70% ethanol at room for 30 min temperature followed by drying at 37 °C for 10–15 min. GelGreen® nucleic acid stain (Biotium, CA, USA) was added onto slides and washed thrice with PBS. Comet formation was observed and recorded under Zeiss Axiovert 200 M microscope (Carl Zeiss Microimaging, Thornwood, NY).

### Real-time quantitative PCR analysis

Control and Mortaparib-treated cells were harvested by trypsin-EDTA (Wako, Japan), and total RNA was extracted using TRIzol™ (Thermo Fisher Sci., Japan). cDNA synthesis was performed using QuantitTech Reverse Transcription Kit (Qiagen, Germany) followed by real-time RT-PCR using SYBR® Green Select Master Mix (Applied Biosystem, Japan). The expression was normalized using 18S as an internal control. Primer sequences used in this study: 18S 5`-CAGGGTTCGATTCCGTAGAG-3` [forward] and 5`-CCTCCAGTGGATCCTCGTTA-3` [reverse], Mortalin 5`- AGCTGGAATGGCCTTAGTCAT-3` [forward] and 5`-CAGGAGTTGGTAGTACCCAAATC-3` [reverse], p53 5`-GTTCCGAGAGCTGAATGAGG-3` [forward] and 5`-TCTGAGTCAGGCCCTTCTGT-3` [reverse], PARP1 5`-TCAGCCTCCTTGCTACAGAGG-3` [forward] and 5`-GGTCGTTCTGAGCCTTTAGGG-3` [reverse]. Single PCR product amplification was confirmed by melt-curve analysis 2^-ΔΔC^T.

### Cell cycle analysis

Control and Mortaparib-treated cells were harvested by trypsin-EDTA, washed with cold PBS, fixed with 70% ethanol on slow vortex and kept at − 20 °C for 12 h. The fixed cells were centrifuged at 3000 rpm at 4 °C for 10 min followed by two washings with cold PBS. Cells were then stained with Guava Cell Cycle Reagent (Merck KGaA, Darmstadt, Germany) in dark for 30 min. RNA was removed by treatment with RNase-A (1 mg/ml; at 37 °C for 30 min) and analysed using Guava® PCA-96 System (Millipore). Cell cycle status was determined by CytoSoft TM Software, version 2.5.6 (Millipore).

### Apoptosis analysis

Cells were seeded in 6-well plate and treated with Mortaparib for 24 h. For each well, media containing floating cells was collected together with harvested cells, and centrifuged at 3000 rpm for 4 °C for 5 min. Cell pellets were re-suspended with 100 μL fresh media and stained with Guava Nexin reagent (EMD Millipore Corporation). Apoptotic cells were quantitated with the help of FlowJo Software (Version 7.6, Flow Jo, LLC, USA).

### PARP1 trapping assay

The inhibition of PARP1 N-terminus was performed using different stringency buffers as follows - Hypotonic buffer: 100 mM MES-NaOH, pH 6.4, 1 mM EDTA, 0.5 mM MgCl_2_, protease inhibitor, 30% sucrose in MiliQ. Buffer 1: 50 mM HEPES-NaOH, pH 7.5, 100 mM KCl, 2.5 mM MgCl_2_, 0.05% Triton X-100, protease inhibitor. Buffer 2: 50 mM HEPES-NaOH, pH 7.5, 250 mM KCl, 2.5 mM MgCl_2_, 0.05% Triton X-100. Buffer 3: 50 mM HEPES-NaOH, pH 7.5, 500 mM KCl, 2.5 mM MgCl_2_, 0.1% Triton X-100, protease inhibitor. Buffer 4: Buffer 1, 5 mM CaCl_2_, MNase.

Cell pellets from control and Mortaparib-treated cells were incubated with hypotonic buffer, centrifuged at 16000 rpm at 4 °C for 10 min. Supernatant was labelled as P1 and pellet was re-suspended with Buffer 1. This step was serially repeated using 1–4 buffers. Supernatant from each washing was labelled as A, B, C and D, respectively. Collected parts were resolved on SDS-PAGE followed by Western blotting with anti-PARP and anti-histone antibodies.

### Immunoprecipitation

Cell lysates containing 500 μg total protein from control and Mortaparib-treated cells were incubated with antibodies or control IgG (Cell Signalling Tech.) at 4 °C for 3 h in slow rotation. A/G PLUS-Agarose beads (Santa Cruz Biotech Inc. sc-2003) were added to the mixture and incubated overnight. Immunoprecipitates were collected by centrifugation at 2500 rpm at 4 °C for 5 min. Pellets were washed 5–6 times with NP-40 buffer followed by centrifugation at 2500 rpm at 4 °C for 5 min. Immunoprecipitates were boiled in SDS sample buffer, resolved on SDS-PAGE and subjected to Western blotting with specific antibodies. For immunoprecipitation assays to determine the binding domain of mortalin to PARP1, COS7 cells (provided high transfection efficiency) were transfected with plasmids expressing V5-tagged deletion mutants of mortalin. Cell lysates were immunoprecipitated with anti-PARP1 antibody, resolved on SDS-PAGE and immunoblotted with anti-V5 antibody.

### Detection of ROS

Cells were cultured on glass coverslips and stained for ROS using Image-IT™ LIVE Green Reactive Oxygen Species (ROS) Detection Kit (Molecular Probes, OR) following the manufacturer’s instructions. ROS expressing cells were photographed using Zeiss Axiovert 200 M microscope and analysed by AxioVision 4.6 software (Carl Zeiss, NY).

### Mitochondrial membrane potential [ΔΨm]

Mitochondrial membrane permeability perturbation was examined using JC-1 staining. Control and Mortaparib-treated cells were stained with JC-1 dye (Ab 141,387) (10 μg/mL) in CO_2_ incubator at 37 °C for 15 min. Cells were washed with PBS and observed under Zeiss Axiovert 200 M microscope (Carl Zeiss, NY).

### Cellular ATP concentration

Total cellular ATP concentration in HeLa cells was determined using a Luminescent ATP Detection Assay Kit (ab113849, Abcam, Cambridge, UK) following the manufacturer’s protocol.

### Caspase-3 activity

Caspase-3 activity in HeLa cells was analyzed using a fluorometric assay kit (Abcam 39,383, Cambridge, MA) following the manufacturer’s protocol.

### Docking and molecular dynamics simulations

The 3D structure of all the proteins was retrieved from Protein Data Bank [Mortalin (4KBO), p53 (1OLG), PARP1-Olaparib complex (5DS3), PARP1-Rucaparib complex (4RV6), PARP1-Niraparib complex (4R6E)] and prepared for docking using protein preparation wizard of Schrodinger. The structure of Mortaparib was prepared using Marvin Sketch and LigPrep. Glide XP docking protocol of Schrödinger was used for all the docking studies. Desmond with Optimized Potential for Liquid Simulations 3 force field was further used to study the dynamic stability of the docked complexes [34, 35].

### Alignment analysis

Known drugs were retrieved from Pubchem database (https://pubchem.ncbi.nlm.nih.gov/) and Mortaparib was drawn using PubChemSketcher structural builder. Alignment and visualization was done using PyMol (Version 2.2.3).

### Antitumor activity of Mortaparib

Female BALB/c nude mice, 4–5 weeks old, were bought from Nihon Clea (Japan). All mice were fed on standard food pellet and water ad libitum, acclimatized to laboratory conditions (24 ± 2 °C), relative humidity of 55–65% and 12 h light/dark cycle for 5–7 days. Ovarian cancer cells (SKOV3) (1 × 10^6^/ml) suspended in PBS were injected subcutaneously (s.c) on left and right flanks of mice (*n* = 4) for subcutaneous model and intraperitoneally (i.p.) for metastasis model. Control and treatment groups were injected (intraperitoneally) with vehicle or Mortaparib (20 mg/kg body weight) for next 20 days, respectively. Mice were weighed and tumor were measured using external calliper every 2 days. Tumor volume was calculated using eq. V = LxW^2^/2, where L is length and W is width. For detection of metastasis, mice were sacrified by cervical dislocation and analysed for the presence of tumors in different tissues.

### Statistical analysis

Statistical data are presented as mean ± SD. All the experiments were performed in triplicate. Unpaired *t* test (GraphPad Prism, GraphPad Software, San Diego, CA) has been performed to determine the degree of significance between the control and experimental samples. Statistical significance was defined as *p* values (*) where * < 0.05, ** < 0.01 and *** < 0.001 represent significant, very significant and very very significant, respectively.

## Results

### Identification of Mortaparib as a p53 activating cytotoxic drug

Mortalin has been established as an oncoprotein that causes cytoplasmic retention and inactivation of p53 tumor suppressor protein in cancer cells. In view of the earlier reports describing differential subcellular distribution of mortalin in normal and cancer cells [8, 10, 11], we screened a library of 12,000 small molecules for their ability to disrupt mortalin-p53 interactions leading to (i) shift in mortalin staining from perinuclear (typical of cancer cells) to pancytoplasmic (typical of normal cells) and (ii) nuclear translocation of p53 (Additional file 1: Figure S1A). Cells treated in 96-well plates were stained for mortalin and p53 followed by automated microscopy. In four rounds of screenings, 6 compounds of which a novel compound was identified and named as Mortaparib. The consequeses of Mortaparib on cells in the forms of mortalin and p53 protien expression was confirmed by immunostaining in a variety of cells. The data demonstrated change in staining pattern of mortalin as well as upregulation and nuclear enrichment of p53 (Additional file 1: Figure S1B and data not shown). We next subjected a variety of cancer cells including ovarian (SKOV3), cervical (HeLa), prostate (DU145 and PC3), non-small cell lung cancer (NCL-H1299), lung cancer (A549), colorectal adenocarcinoma (DLD1), pancreatic carcinoma (SUIT-2), hepatoma (PLC) and human gastric (MKN-45) to 5 μM Mortaparib and found some cell lines to be more responsive than others. Amongst all, HeLa and SKOV3 showed the highest cytotoxicity (Additional file 1: Figure S1C). Dose-dependent cytotoxic analyses endorsed Mortaparib to be toxic in the range of 2–5 μM in HeLa, ME180, SKG-II and SKG-IIIb (cervical cancer), SKOV-3 and OVK18 (ovarian cancer) cells (Additional file 1: Figure S1D). Long term viability assay showed dose dependent decrease in colony forming efficacy at lower doses of Mortaparib (Additional file 1: Figure S1E). In view of this data, we selected Mortaparib for further analysis. Structure of Mortaparib (5-[1-(4-methoxyphenyl)(1,2,3,4-tetraazol-5-yl)]-4-phenylpyrimidine-2-ylamine) is shown in Additional file 1: Figure S1F.

Based on the above data, we were prompted to investigate the structural homology of Mortaparib to drugs currently being used for cervical and ovarian cancers. As shown in Additional file 1: Figure S2A, analysis using structural alignment viewed by PyMol revealed that Mortaparib shares no similarity with currently known drugs. Furthermore, we used Olaparib, the PARP1 targeting drug used for ovarian cancer, and compared the response of cells to Mortaparib and Olaparib. Interestingly, we found similar cytotoxicity profile of the two drugs. Of note, SKOV3 cells showed higher toxicity in response to Mortaparib than Olaparib in several independent experiments (Additional file 1: Figure S2B). Since Mortaparib was identified as an abrogator of mortalin-p53 interactions, at first we computationally explored its potential to bind with mortalin and p53. The docking score with mortalin and p53 was found to be − 3.338 and − 2.477 kcal/mol respectively. Though the score was low, Mortaparib showed binding with the p53-interacting residues in mortalin (Additional file 1: Figure S2C and D). In order to further investigate the binding interactions of mortalin and Mortaparib, the complex was simulated in explicit water model for 100 ns. As shown in Additional file 1: Figure S3A and B, we found that Mortaparib did not interact stably at any single site of mortalin and hence the cytotoxicity in cells might not be the result of direct abrogation of mortalin-p53 interaction by Mortaparib.

### Mortaparib-treated cells showed p53-mediated growth arrest and apoptosis

**Fig. 1:**
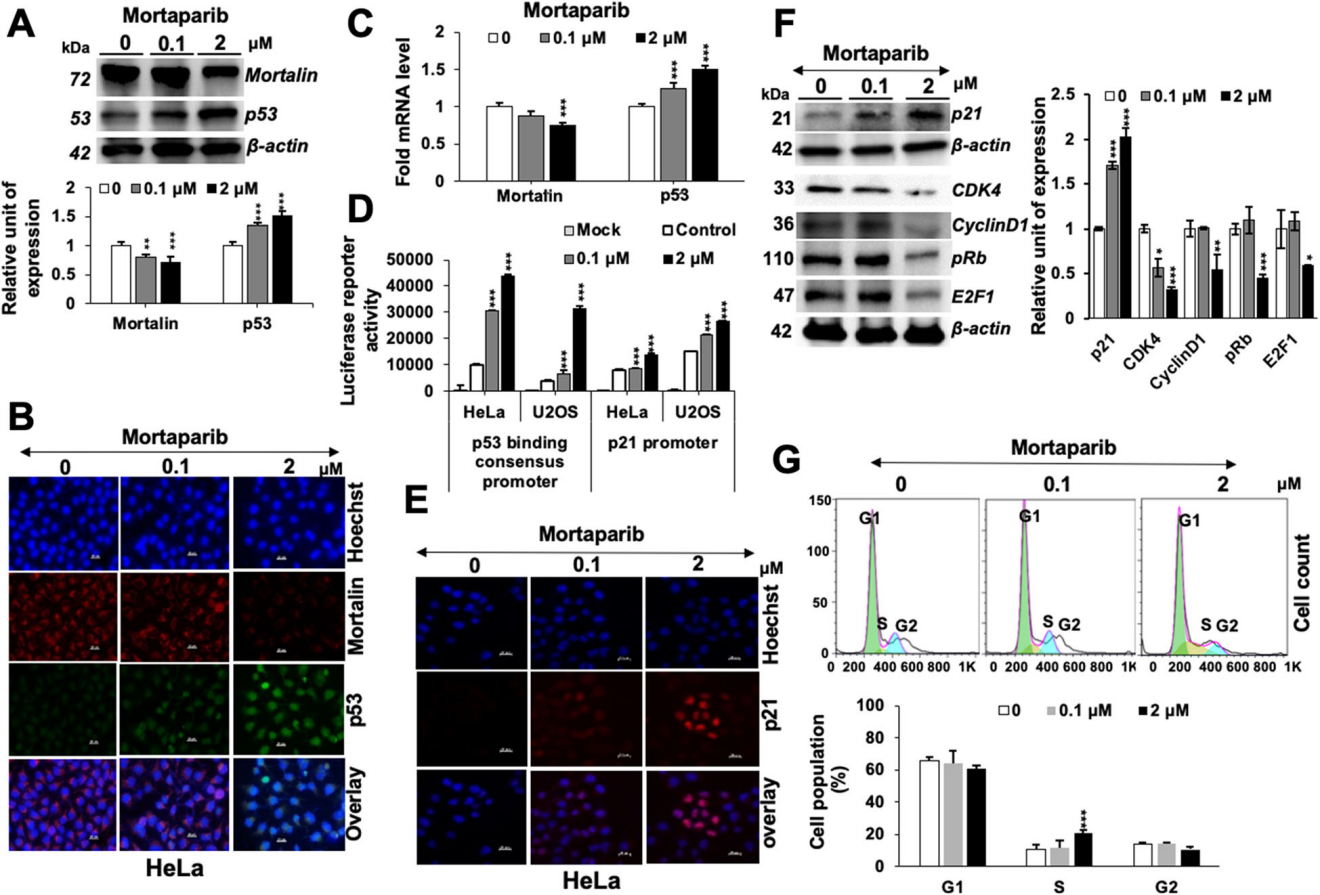
Mortaparib targets mortalin and results in growth arrest in HeLa cells. Control and Mortaprib-treated cells were analyzed for the expression level of mortalin and p53 by Western blotting (**a**), immunostaining (**b**) and RT-PCR (**c**). Decrease in mortalin and increase in p53 levels was marked in all assays. p53-specific luciferease reporter assays using either p53-binding consensus sequence or p21-promoter (~ 2.8 kb) showed increase in p53-driven luciferase in Mortaparib-treated HeLa as well as U2OS cells (**d**). Mortaparib-treated cells showed increase in p21 by immunostaining (**e**). Western blot analysis showed increase in p21 and decrease in CDK4, Cyclin D1, pRb and E2F1. Quantitation is shown on the right (**f**). Cell cycle analysis showed increase in number of HeLa cells in S phase upon Mortaparib treatment (**g**). The quantitative data represents mean ± SD obtained from, at least, three independent experiments; *p*-values were calculated using Student’s *t*-test. * < 0.05, ** < 0.01 and *** < 0.001 represent significant, very significant and very very significant, respectively. Scale bar in B and E = 20 μM

**Fig. 2:**
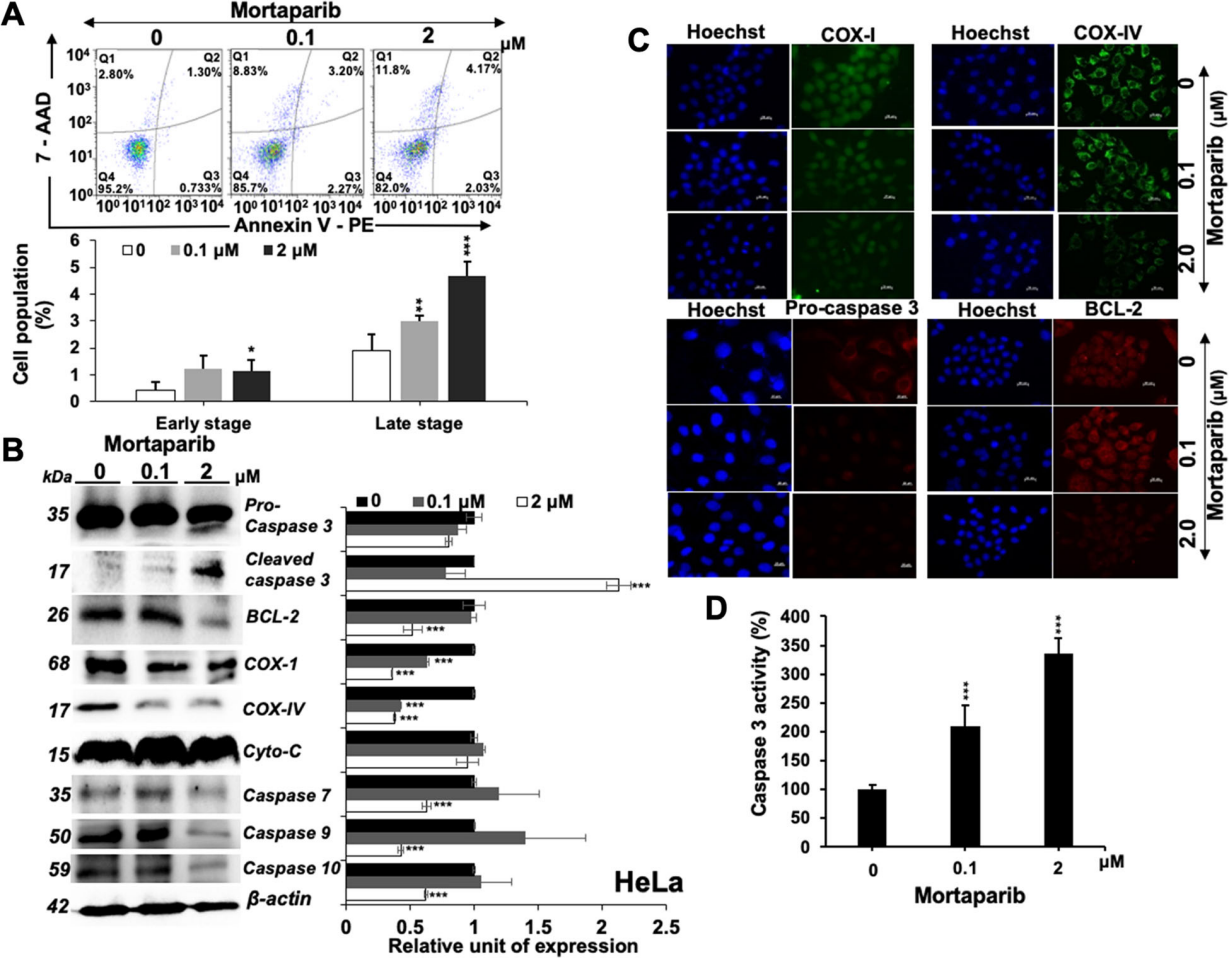
Mortaparib-treated HeLa cells undergo apoptosis. Flow cytometric analysis showing increase in apoptotic cells in dose dependent manner following Mortaprib treatment (**a**). Western blot analysis showing molecular markers of apoptosis in control and Mortaparib-treated cells (**b**). Decrease in pro-caspase, BCl2, COX I, COX IV, Caspase-7, − 9, − 10 in Mortaparib-treated cells was recorded. Cleaved caspase-3 showed increase. Immunostaining showed decrease in COX I, COX IV, Procaspase 3 and BCl2 proteins in Mortaparib-treated cells (Scale bar = 20 μM). **c**. Caspase-3 activity, as evaluated using a fluoremetric assay, showed increase in dose-dependent manner (**d**). The quantitative data represents mean ± SD obtained from, at least, three independent experiments; *p*-values were calculated using Student’s *t*-test. * < 0.05, ** < 0.01 and *** < 0.001 represent significant, very significant and very very significant, respectively

We next determined the expression of mortalin and p53 in control and Mortaparib-treated cells. As shown in Fig. 1a, Mortaparib-treated cells showed upregulation of p53, endorsed by immunostaining (Fig. 1b). Of note, we found that mortalin was downregulated in Mortaparib-treated cells, at protein as well as mRNA levels (Fig. 1a-c). Wild type p53-dependent luciferase reporter assays endorsed upregulation of p53 (Fig. 1d), and was further confirmed by upregulation of p21^WAF-1^, downstream growth-arrest mediating effector of wild type p53 (Fig. 1e and f) and subsequent decrease in CDK4, CyclinD1, pRb and E2F1 proteins (Fig. 1f) signifying growth arrest. Cell cycle analysis endorsed an arrest of Mortaparib-treated cells in S phase (Fig. 1g). On the other hand, analyses of control and treated cells also revealed increase in the number of cells in apoptosis (Fig. 2a). We, therefore, next analyzed the expression of key regulators of apoptosis. As shown in Fig. 2b, consistent with the induction of apoptosis, Mortaparib-treated cells showed decrease in anti-apoptotic proteins: Bcl-2, COX-1, COX-IV and Procaspase-3, − 7 and − 9 by Western blotting. Cleaved Caspase-3, on the other hand, showed an increase (Fig. 2b). The results were confirmed by immunostaining (Fig. 2c and data not shown) and Caspase 3 activity assays (Fig. 2d).

### Mortaparib-treated cells showed inhibition of PARP1 and activation of DNA damage signaling

Since Mortaparib mimicked the cytotoxic profile of Olaparib, an established inhibitor of PARP1 [23], we next looked into molecular interactions of Mortaparib with PARP1 protein. PDB structure of PARP1 co-crystallized with Olaparib, Rucaparib and Niraparib were used as reference. We found that Mortaparib and all the three known inhibitors were docking at the same site of PARP1. The docking score of Mortaparib was − 6.28 kcal/mol. The Mortaparib-PARP1 docked complex was then simulated in explicit water for 100 ns to account for the binding stability. Additional file 1: Figure S4 illustrates the binding orientation of Mortaparib, Olaparib, Rucaparib and Niraparib along with the interacting residues of PARP1. All the compounds were binding in the same cavity lined by the catalytically active residues of PARP1 (662–1014 aa). Mortaparib was also interacting with the residues forming the main signature motif (859–908 aa) of PARP1 showing binding behavior similar to that of the known PARP1 inhibitors.

**Fig. 3:**
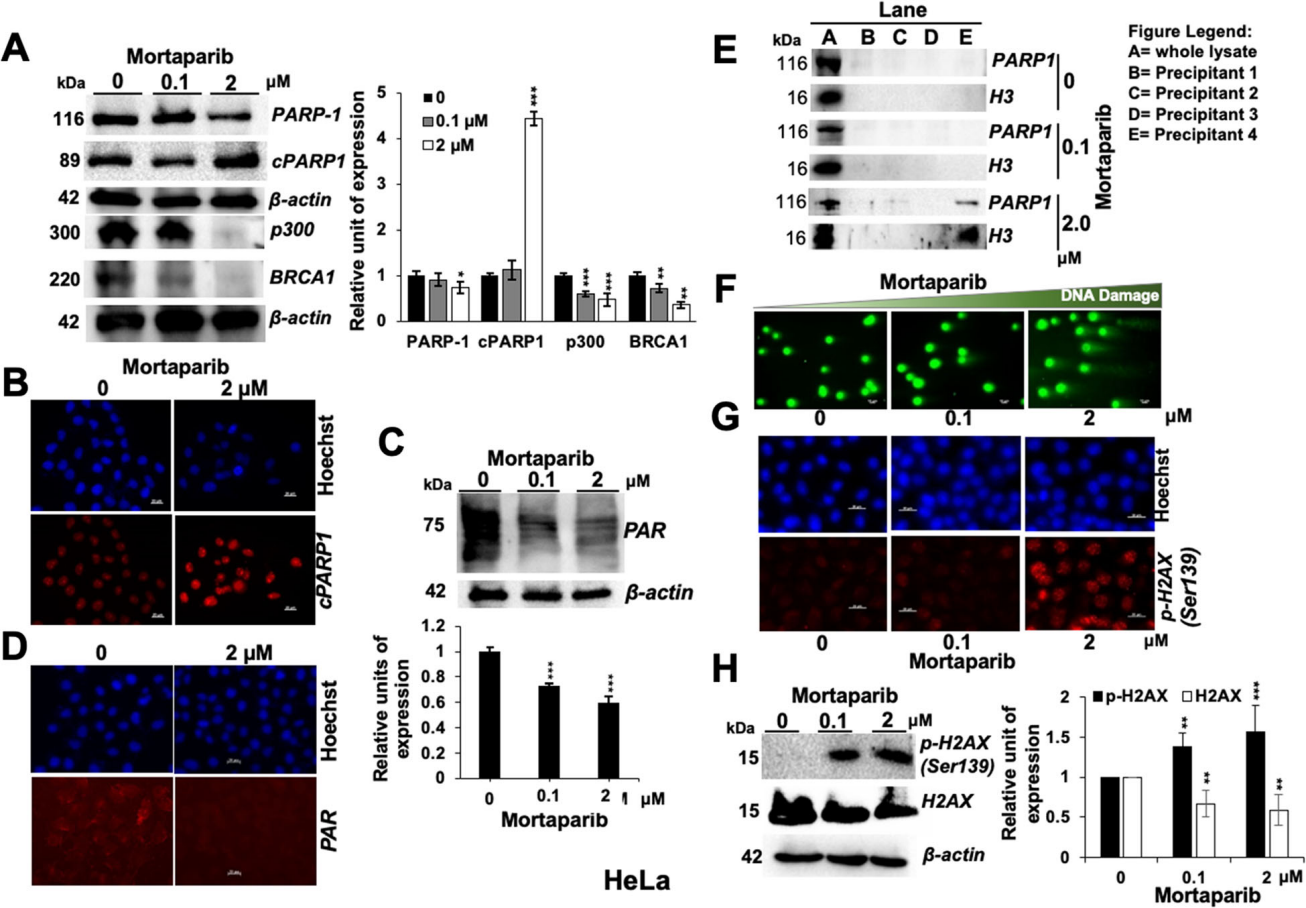
Mortaparib caused inhibition of PARP1 and activation of DNA damage stress signaling in HeLa cells. Western blot analysis showed Mortaparib treatment caused downregulation of PARP1, p300 and BRCA1 and increase in cleaved PARP1 (**a**). Immunostaining revealed increase in cleaved PARP1 (Scale bar = 20 μM) (**b**) and decrease in PAR by Western blot (**c**) and immunostaining in Mortaparib-treated cells (**d**). Western blot analysis of PARP1-DNA complexes showed trapping of PARP1 in DNA in Mortaparib (2 μM) treated cells (**e**). Neutral comet assay showing Mortaparib induced increase in DNA damage (Scale bar = 10 μM) (**f**). Immunostaining of γH2AX in control and Mortaparib-treated cells showing increase in the number of γH2AX foci in the latter (Scale bar = 20 μM) (**g**). Western blot showed Mortaparib treatment led to increase in γH2AX (**h**). The quantitative data represents mean ± SD obtained from, at least, three independent experiments; *p*-values were calculated using Student’s *t*-test. * < 0.05, ** < 0.01 and *** < 0.001 represent significant, very significant and very very significant, respectively

**Fig. 4:**
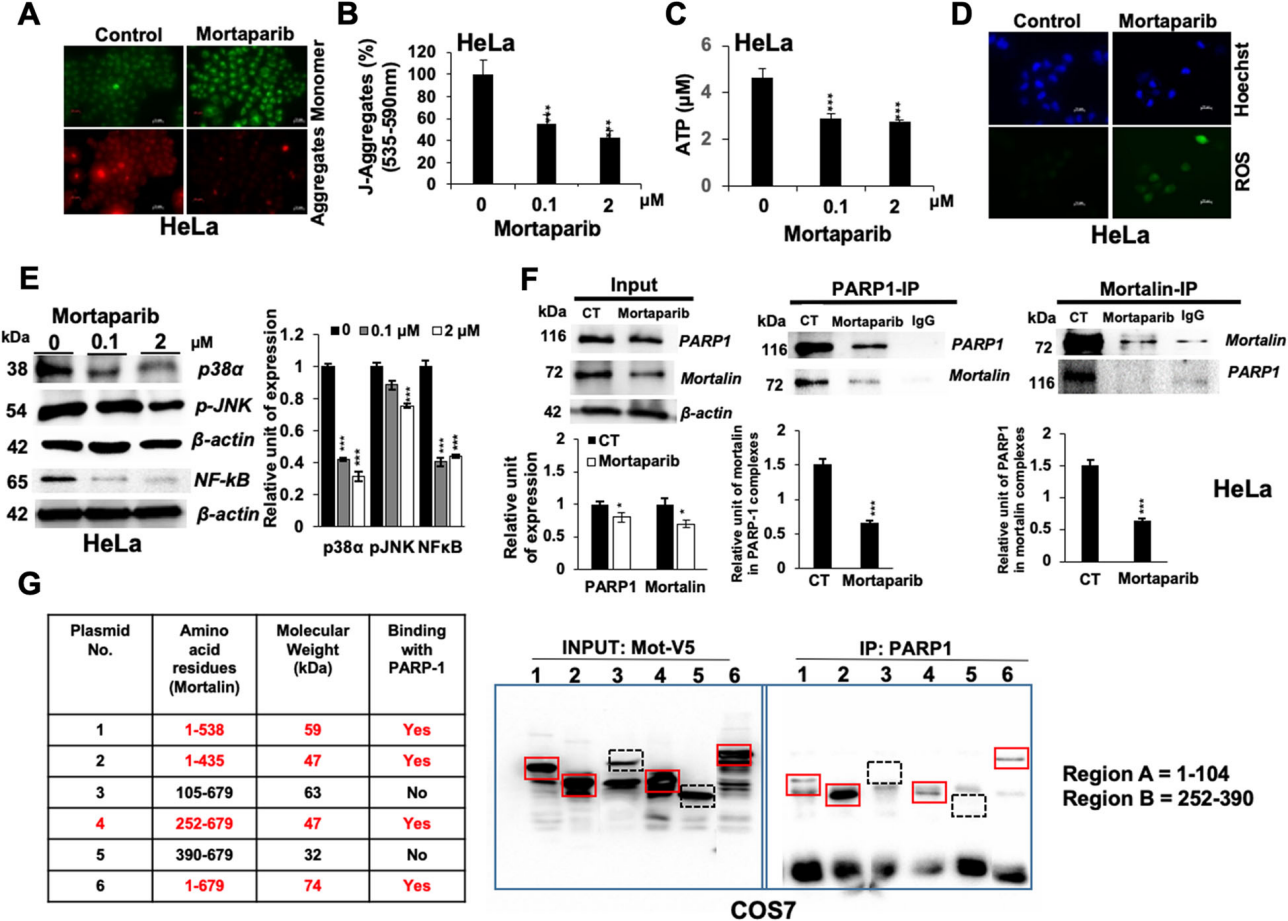
Mortaparib treatment caused oxidative stress and targeted mortalin-PARP1 interactions. JC-1 staining showed lower mitochondrial membrane poteintial as signified by increase in its monomers and decrease in aggregates in Mortaparib-treated HeLa cells (Scale bar = 20 μM); microscopic images (**a**) and their spectrophotometeric analysis (**b**) are shown. Mortaparib treatment showed decrease in total cellular ATP concentration (**c**) and increase in ROS in HeLa cells (Scale bar = 20 μM) (**d**). Western blot analysis of control and Mortaparib-treated cells showing decrease in p38, p-JNK and NF-κB; Quantitation is shown on the right (**e**). Co-immunoprecipitation of PARP1-mortalin (**f**) revealed that they exist in a complex, and Mortaparib abrogated their interaction. PARP1 interacting domain of mortalin was defined by immunoprecipitation of the two proteins from lysates of COS7 cells transfected with mortalin deletion mutants tagged with V5 (**g**). Deletion mutants (amino acids residues shown in the Table) that showed binding with PARP1 are marked with red squares. The ones that did not bind to PARP1 are marked by dotted black squares. Mortalin-PARP1 interactions were assigned to two domains (i) amino acid residues 1–104 and (ii) 252–390. The quantitative data represents mean ± SD obtained from, at least, three independent experiments; *p*-values were calculated using Student’s *t*-test. * < 0.05, ** < 0.01 and *** < 0.001 represent significant, very significant and very very significant, respectively

We next examined PARP1 in control and Mortaparib-treated cells. As shown in Fig. 3, the latter showed decrease in both PARP1 (116-kDa) and PAR, increase in cleaved PARP1 protein (89-kDa) by Western blotting and immunostaining (Figs. 3a-d). PARP1 has been reported to directly interact with histone acetyl-transferase (p300) and NF-kB and regulate inflammation and oxidative stress signaling [36]. Inactivation of BRCA1 (breast cancer and ovarian cancer-specific tumor suppressor protein) by mutations or promoter methylation has been shown to be associated with upregulated PARP1. Overexpression of BRCA1, on the other hand, was shown to downregulate PARP1 [37]. PARP1 inhibitors suppressed BRCA1 or BRCA2 afflicted tumors through synthetic lethality [38] and therefore deemed beneficial for management of BRCA1 mutated tumors [39]. In this premise, we examined the status of p300 and BRCA-1 proteins in Mortaparib-treated cells and found significant decrease of both (Fig. 3a). Furthermore, by DNA trapping assay [25, 30], we found that PARP1 was trapped in DNA in Mortaparib-treated cells that signified decrease in PAR and inactivation of single strand DNA repair resulting in accumulation of DNA damage (Fig. 3e). The latter was also endorsed by DNA comet assay (Fig. 3f), γH2AX staining (Fig. 3g) and Western blotting (Fig. 3h) signifying accumulation of double strand DNA breaks. Since ATP generated in mitochondria is an essential component of PARP1 function [40, 41], we determined if Mortaparib induced inactivation of PARP1 was mediated by decrease in ATP and aberrant mitochondrial function due to decrease in mortalin [42]. Indeed, analysis of mitochondrial membrane potential and ATP revealed decrease in membrane potential and ATP level, and increase in ROS level in Mortaparib-treated cells (Figs. 4a-d). Consistent with the oxidative stress phenotype, Mortaparib-treated cells showed decrease in p38, p-JNK and NFk-B (Fig. 4e) signifying activation of growth arrest/apoptosis signaling.

### Mortaparib targeted mortalin-PARP1 interactions

Mortalin has been shown to be a dynamic protein localized at multiple subcellular sites including mitochondria, nucleus, ER and plasma membrane [43]. Although PARP1 is conventionally treated as a nuclear factor important for single strand DNA repair, several studies in the past have shown its occurrence in mitochondria [44, 45]. ADP-ribosyl transferase (ART), required for PARP1 function, was also shown to be present intact in submitochondrial fractionations [45]. In view of this information and our data on the effect of Mortaparib on mortalin and PARP1, we predicted that these two proteins may interact either in nucleus and/or in mitochondria. Bioinformatics analysis revealed that mortalin and PARP1 possess potent interaction domains (Additional file 1: Figure S5A). In addition, mortalin-interacting region of PARP1 was close to the one that interacted with Mortaparib (Additional file 1: Figure S5A and B). The amino acid residues 71–419 of PARP1 were predicted to be involved in its binding to mortalin. On the other hand, amino acid residues 42–334 of mortalin interacted with PARP1. Mortaparib was predicted to bind to the carboxy terminus of PARP1 (662–1014 aa) that was outside the direct binding region of PARP1 to mortalin. In order to test these predictions, we next performed co-immunoprecipitation of PARP1 and mortalin, and found that PARP1-immunocomplexes contained mortalin and vice versa (Fig. 4f) demonstrating that these two proteins interact in cells. Of note, Mortaparib-treated cells showed decrease in both proteins and more so in their interaction as judged from immunocomplexes (Fig. 4f). In order to validate the interactions further, we used COS7 cells expressing V5-tagged deletion mutants of mortalin. Immunoprecipiation with anti-PARP1 antibody revealed co-immunoprecipiation of V5-tagged deletion mutants of mortalin protein endorsing their interaction. Of note, some delection mutants did not co-precipiate and revealed that mortalin interacts with PARP1 by amino acid residues present in two domains (i) 1–104 and (ii) 252–390 (Fig. 4g).

### Mortalin overexpression and silencing affected PARP1 signaling

**Fig. 5:**
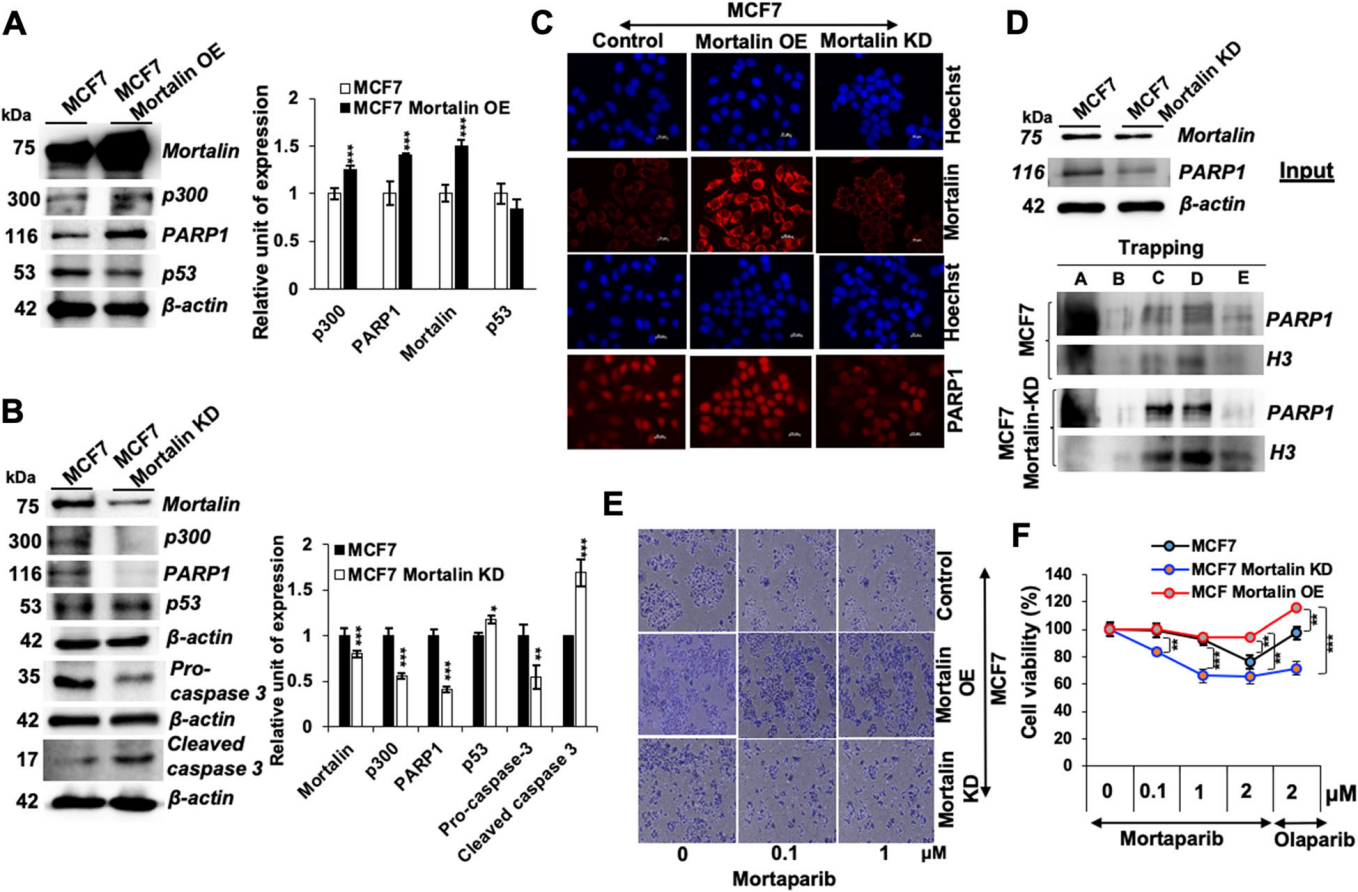
Mortalin treatment, overexpression and knockdown modulates PARP1 signaling. Western blots showing increase in p300, PARP1 and decrease in p53 levels in mortalin-overexpressing MCF7 cells; quantitation is shown on the right (**a**). Mortalin-compromised cells showed decrease in p300, PARP1 and Pro-caspase 3; p53 and cleaved caspase showed increase in the same cells; quantitation is shown on the right (**b**). Immunostaining also showed increase in PARP1 in mortalin-overexpressing cells and decrease in mortalin-knockdown cells (Scale bar = 20 μM) (**c**). PARP1-DNA trapping assay revealed trapping of PARP1 in DNA in mortalin-compromised cells. Upper panel shows the input. The lower panel showed trapping assay; histone H3 was used as a loading control (**d**). Mortalin-overexpressing and knockdown cells showed resistance and sensitization to Mortaparib, repectively, as determined by Crystal violet staining (**e**) and cell viability assay (**f**). The quantitative data represents mean ± SD obtained from three independent experiments; *p*-values were calculated using Student’s t-test. * < 0.05, ** < 0.01 and *** < 0.001 represent significant, very significant and very very significant, respectively

In order to further support that mortalin-PARP1 interactions may be functionally relevant, we recruited overexpression and knock-down of mortalin and determined PARP1 activities (Fig. 5). In order to overcome the effect of human papilloma virus (present in HeLa cells) on p53 activity, we used MCF7 cells for this assay. As shown in Fig. 5a, mortalin overexpressing cells, as expected, showed decrease in p53. Of note, increase in PARP1 was observed in these cells (Fig. 5a). On the other hand, mortalin compromised cells showed decrease in PARP1, increase in p53 and decrease in pro-caspase 3 (Fig. 5b). The data was confirmed by immunostaining with specific antibodies (Fig. 5c). Furthermore, similar to Mortaparib-treated cells, mortalin shRNA-treated cells showed trapping of PARP1 (Fig. 5d). We also found that whereas cells overexpressing mortalin showed resistance to Mortaparib, mortalin-compromised cells were significantly sensitized to Mortaparib (Fig. 5e and f). These data strongly supported that Mortaparib-induced inhibition of PARP1 signaling and apoptosis in cancer cells was mediated by targeting of mortalin.

### Mortaparib caused inhibition of cell migration, invasion and angiogenesis in vitro and tumor suppression in vivo

**Fig. 6:**
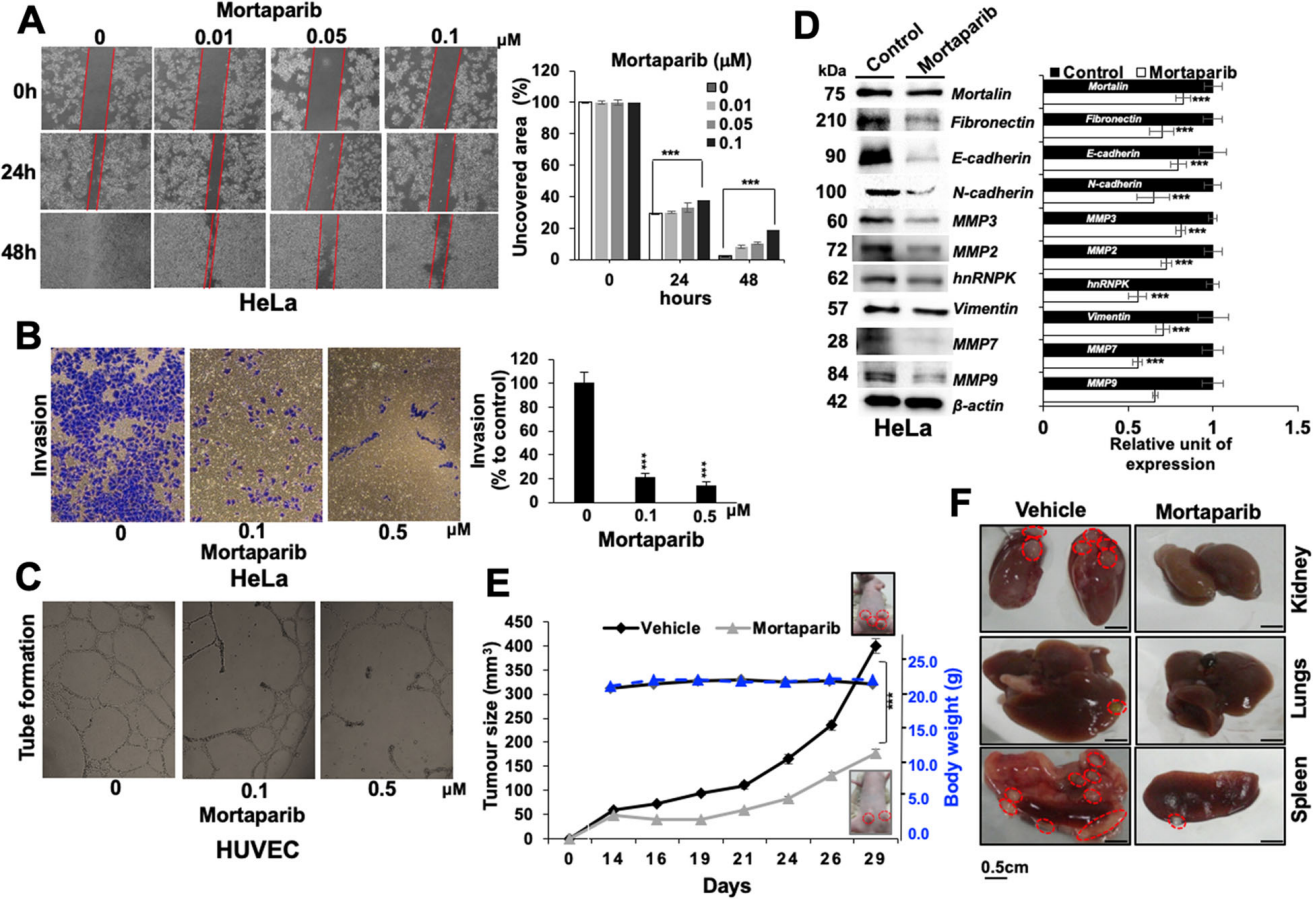
Mortaparib treatment caused inhibition of cell migration, invasion and angiogenesis in in vitro and tumor suppression in vivo. Mortaparib treatment showed delay in migration (**a**), invasion (**b**) in HeLa cells and tube formation (**c**) in HUVEC cells. Western blot showed decrease in metastasis markers including mortalin, fibronectin, N-cadherin, MMP3, MMP2, MMP7, MMP9, hnRNPK and vimentin in Mortaparib-treated cells (**d**). Data represents mean ± SD from, at least, three independent experiments; *p*-values were calculated using Student’s t-test. * < 0.05, ** < 0.01 and *** < 0.001 represent significant, very significant and very very significant, respectively. Mortaparib-treated mice showed decrease in SKOV3 tumor volume with no change in body weight. Representative tumors in control and Mortaparib-treated mice (at day 29) are shown (**e**). Metastasis of cells to stomach, kidney, lung and spleen was inhibited in Mortaparib-treated mice (F)

In light of the information that overexpression of mortalin and PARP1 [5, 21, 46] promotes cell migration, invasion and angiogenesis, we next asked if inhibition of mortalin and PARP1 by Mortaparib was sufficient to block these phenotypes of cancer cells. Mortaparib-treated cells showed remarkable dose-dependent inhibition of cell migration (analysed by Wound-scratch assay) (Fig. 6a), cell invasion (analysed by Boyden Chamber Assay) (Fig. 6b) and angiogenesis (analysed by tube formation assay using HUVEC cells) (Fig. 6c). Consistent with these phenotypes, molecular analyses showed down-regulation of proteins involved in epithelial-mesenchymal transition (EMT). These included fibronectin, N-Cadherin, MMP2, MMP3, MMP-7, MMP-9, hnRNPK and vimentin in Mortaparib-treated cells (Fig. 6d); E-cadherin, however, showed decrease. Finally, we determined the in vivo tumor suppressor efficacy of Mortaparib. As shown in Fig. 6e and f, Mortaparib-treated (20 mg/kg body weight) mice showed suppression of tumor growth in subcutaneous xenografts of SKOV-3 cells; and was not toxic to mice. In metastasis model, control but not the Mortaparib-treated mice showed remarkable tumors in kidney, lungs and spleen.

## Discussion

Mortalin is a stress chaperone that has been implicated in human carcinogenesis. In cancer cells enriched with mortalin expression, it has been shown to inactivate p53 tumor suppressor protein and activate others including telomerase and hnRNPK that promote cancerous properties [10]. Mortalin knockdown has earlier been reported to cause growth arrest, apopotosis, reversal of EMT and cancer cell stemness [5, 7]. Hence, mortalin-targeting drugs have been deemed useful for cancer therapy. Differential subcellular distribution of mortalin in normal and cancer cells has been reported [43, 47]. Furthermore, several studies showed that mortalin interacts with p53 in cancer cells, causing its cytoplasmic retention and inactivation of tumor suppressor activity [8, 9]. Small molecules that interfere with mortalin-p53 interaction, including MKT-077, Withaferin A and CAPE and specific anti-mortalin molecules (ribosomes, shRNA) were shown to activate p53 and resulting in growth arrest of cancer cells [12, 48–52]. In view of these reports, we established a screening assay to visually detect activation of p53 (nuclear translocation) associated with shift in mortalin staining from perinuclear (cancer cells) to pancytoplasmic (normal cells) and screened a library of 12,000 small molecules. Mortaparib was selected as a strong candidate after four rounds of screening (Additional file 1: Figure S1A and B). It showed cytotoxicity to a variety of cancer cells of which HeLa and SKOV3 showed strong response in many independent experiments (Additional file 1: Figure S1C and D). At molecular level, we found that Mortaparib, not only leads to abrogation of mortalin-p53 interaction, nuclear translocation and activation of p53 activity but also downregulates mortalin at the transcriptional level. Mortalin has been reported for several functions that are essential for cell survival, maintainance of mitochondrial integrity, ATP generation and chaperoning [7]. Indeed, we found that Mortaparib treatment caused depolarization of mitochondria membrane, ROS activation and decrease in cellular ATP levels (Figs. 3 and 4). The latter is an important energy substrate and co-factor involved in post-translational modification (polyADP-ribosylation) and activation of PARP1, an essential step for PARP1-mediated DNA repair genomic stability and cell proliferation. PARP1 is often enriched in cancer cells and its inhibition has been shown to cause cell death [53] making it an attractive target for cancer treatment. In recent years, PARP1 inhibitors have extensively been explored as anticancer drugs either as single agent or in combination with other conventional chemotherapeutic drugs for treatment of breast and ovarian cancers [54]. We next determined the effect of Mortaparib on PARP1 signaling. Indeed, Mortaparib-treated cells showed reduced PARP1 function, decrease in PAR and its trapping into DNA, resulting in accumulation of DSB (Figs. 3 and 5). In cell viability assays, Mortaparib showed cytotoxicity profile similar to that of Olaparib in various cell lines (Additional file 1: Figure S2). In order to investigate the molecular link between mortalin and PARP1, we recruited mortalin overexpressing and compromised cells, and found an association between the two proteins. Whereas mortalin-overexpressing cells showed activated PARP1 signaling, its knockdown lead to decrease in PARP1 level as well as its downstream regulators evoking apoptosis. Furthermore, mortalin-compromised cells were found more sensitive to the Mortaparib treatment, as compared to overexpression derivatives (Fig. 5). By co-immunoprecipitation, we found that PARP1 and mortalin proteins interact and these interactions are targeted by Mortaparib, as supported by molecular docking and experimental data (Additional file 1: Figures. S4 and S5). p53 has been shown to interact with multiple proteins. Besides its interaction with PARP1, it has been identified as a substrate for covalent and non-covalent interaction with PAR [55, 56]. Central and carboxy terminal region of p53 were found essential for interaction and complex formation with PARP1. Interestingly, we have previously reported that mortalin (residues 253–282) binds to the carboxy terminal amino acid residues 312–352 of p53 [9]. Co-immunoprecipitation of mortalin and PARP1, using the deletion mutants of mortalin, demonstrated that mortalin binds to PARP1 by two domains (amino acid residues 1–104 and 252–290). One of these includes p53 binding region. Taken together, these data suggested that Mortalin, p53 and PARP1 make tricomplex that may be abrogated by Mortaparib leading to functional activation of p53 and inactivation of PARP1. Indeed, Mortalin and PARP1 targeting by Mortaparib caused growth arrest and apoptosis of cancer cells, marked by activation of p53-p21^WAF1^ and caspase signalings, respectively. Oxidative stress, depolarization of mitochondrial membrane and decrease in ATP signified apoptosis of cells [57, 58].

Sub-toxic doses of Mortaparib showed significant inhibition of cell migration, invasion and angiogenic abilities in vitro as well as growth and metastasis in vivo (Fig. 6) suggesting it to be a potent anti-tumor and anti-metastasis drug. Cells treated with Mortaparib showed decrease in N-Cadherin, MMP3, MMP2, MMP7, MMP9 and hnRNP-K. However, Mortaparib-induced decrease in cell migration, invasion and angiogenesis seems not to involve E-Cadherin as it remained unchanged. γH2AX is known as sensitive indicator of DNA double strand break and genotoxic stress. BRCA1 plays a major role in homologous recombination repair pathway and its knockdown results in steady increase in the level of γH2AX due to genotoxic stress [59]. Of note, targeting mortalin and PARP1 by Mortaparib also caused decrease in BRCA1 that has earlier been shown to cause collateral lethality of cancer cells [38] and increase in γH2AX protein. Ovarian and breast tumors with BRCA1 and BRCA2 mutations are sensitive to the PARP1 inhibitors [39]. We found that Mortaparib-treated cells show remarkable decrease in BRCA1 and activated DNA damage signaling as signified by increase in γH2AX protein expression. These effects are likely to be mediated by inactivation of PARP1 and activation of p53 signaling by Mortaparib. Recently, Olaparib has been approved as the first PARP inhibitor in European Union and United States for the treatment of advanced BRCA-mutated ovarian cancer [60]. Mortaparib may be another candidate drug for these cancers. Taken together, further studies are warranted to understand the molecular mechanisms of anticancer activity of Mortaparib and its clinical efficacy.

## Conclusion

Mortaparib inhibits mortalin and PARP1 resulting in activation of growth arrest and apoptosis signaling in cancer cells in vitro and in vivo. To the best of our knowledge this is the first report describing a compound (hereby named as Mortaparib) that shows dual inhibition of mortalin and PARP1 and may offer better outcome in cancer chemotherapy and awaits clinical trials.

## Supplementary information

**Additional file 1: Figure S1.** Drug screening and identification of Mortaparib as a p53-activating drug. **Figure S2.** Identification of Mortaparib as a new. **Figure S3**. RMSD and different docking poses of Mortaparib with mortalin. **Figure S4.** Molecular docking showinginteractions of Mortaparib and PARP1. **Figure S5.** Molecular docking showing interactions of PARP1 and Mortalin is shown (A) ; interacting residues of the two proteins are listed in the table on the right (B). Coimmunoprecipitation of PARP1 and Mortalin in MCF7 showing inputs of the two proteins (a), presence of mortalin in PARP1 immunocomplexes (b) and vica versa (c). The quantitative data represents mean ± SD obtained from at least three independent experiments; *P*-values were calculated using Student’s t-test. *< 0.05, **< 0.01, and ***< 0.001 represent significant, very significant, and very very significant, respectively.

## Data Availability

The datasets used and/or analyzed during the current study are available within the manuscript and its supplementary information files.

## Abbreviations

ATM: Ataxia telangiectasia mutated
ATP: Adenosine triphosphate
ATR: Ataxia telangiectasia and Rad3 related
BRCA1: Breast cancer susceptibility gene 1
CHK1: Checkpoint kinase 1
CHK2: Checkpoint kinase 2
DNA: Deoxyribonucleic acid
DSB: Double strand break
EMT: Epithelial to mesenchymal transition
GRP75: Glucose regulated protein 75
hnRNPK: Heterogenous nuclear ribonucleo protein K
NAD^+^: Nicotinaminde adenine dinucleotide
PARG: Poly(ADP-ribose) glycohydrolase
PARP1: Poly [ADP-ribose] polymerase 1
PDB: Protein data bank
shRNA: Short hairpin Ribocucleic acid
siRNA: Small interfering RNA
