# Supplementary material for: Mortaparib, a novel dual inhibitor of mortalin and PARP1, is a potential drug candidate for ovarian and cervical cancers
Source: J Exp Clin Cancer Res. 2019 Dec 19;38:499. doi: 10.1186/s13046-019-1500-9 (PMC6923857; doi:10.1186/s13046-019-1500-9)
Supplement: Supplementary file 1 — Additional file 1: Figure S1. Drug screening and identification of Mortaparib as a p53-activating drug. Figure S2. Identification of Mortaparib as a new. Figure S3. RMSD and different docking poses of Mortaparib with mortalin. Figure S4. Molecular docking showinginteractions of Mortaparib and PARP1. Figure S5. Molecular docking showing interactions of PARP1 and Mortalin is shown (A) ; interacting residues of the two proteins are listed in the table on the right (B). Coimmunoprecipitation of PARP1 and Mortalin in MCF7 showing inputs of the two proteins (a), presence of mortalin in PARP1 immunocomplexes (b) and vica versa (c). The quantitative data represents mean ± SD obtained from at least three independent experiments; P-values were calculated using Student’s t-test. *< 0.05, **< 0.01, and ***< 0.001 represent significant, very significant, and very very significant, respectively. [file 13046_2019_1500_MOESM1_ESM.pdf]

SUPPLEMENTARY INFORMATION

Mortaparib, a novel dual inhibitor of mortalin and PARP1, is a potential drug candidate for ovarian and cervical cancers

Jayarani F. Putri[1], Priyanshu Bhargava[1], Jaspreet Kaur Dhanjal[1,2],
Tomoko Yaguchi[1], Durai Sundar[2], Sunil C. Kaul[1*] and Renu Wadhwa[1*]

[1]DBT-AIST International Laboratory for Advanced Biomedicine [DAILAB], DAICENTER, National Institute of Advanced Industrial Science & Technology [AIST], Tsukuba - 305 8565, Japan

[2]DAILAB, Department of Biochemical Engineering & Biotechnology, Indian Institute of Technology [IIT] Delhi, Hauz Khas, New Delhi - 110 016, India.

Running Title: *A novel dual inhibitor of mortalin and PARP1*

*Correspondence: renu-wadhwa@aist.go.jp; s-kaul@aist.go.jp

National Institute of Advanced Industrial Science & Technology (AIST), Central 5, Higashi 1-1-1, Tsukuba, Ibaraki- 305 8565, Japan.

Additional files

Additional file 1: Supplementary Figure 1 (S1). Drug screening and identification of Mortaparib as a p53-activating drug. Schemetic representation of screening assay (A). Double immunostaining of mortalin and p53 in MCF7 and U2OS showed that Mortaparib treatment caused shift in mortalin staining pattern from perinuclear to pancytoplasmic, and increase in nuclear p53 (Scale bar=5μM) (B). Cell viability assay showing different level of cytotoxicity of Mortaparib to various cancer cell lines (C). Dose-dependent cytotoxicity was detected in HeLa, ME180, SKG-II, SKG-IIIb, OVK18, SKOV3; the latter two of these showed remarkably stronger effect as compared to other cancer cells and the normal fibroblasts (MRC5) (D). Long-term viability assay (colonogenicity) showed decrease in the number of colonies in Mortaparib-treated HeLa cells (E). Chemical structure of Mortaparib and its molecular weight (F). The quantitative data represents mean ± SD obtained from at least three independent experiments; *p*-values were calculated using Student's *t*-test. *< 0.05, **< 0.01, and ***< 0.001 represent significant, very significant, and very very significant, respectively.

Additional file 2: Supplementary Figure 2 (S2). Identification of Mortaparib as a new drug. Structural homology of Mortaparib with several known drugs clinically used for treatment of ovarian and cervical cancer is shown (A). Cell viability assay with Mortaparib showed that it mimics Olaparib activity (a conventional drug for ovarian cancer treatment) for HeLa, U2OS, MCF7, A549, H1299 cells and was remarkably cytotoxic to SKOV3 cells (B). The quantitative data represents mean ± SD obtained from at least three independent experiments; *p*-values were calculated using Student's *t*-test. *< 0.05, **< 0.01, and ***< 0.001 represent significant, very significant, and very very

significant, respectively. Molecular docking of Mortaparib with mortalin (C) and p53 (D); Mortaparib did not show significant binding to Mortalin.

Additional file 3: Supplementary Figure 3 (S3). RMSD and different docking poses of Mortaparib with mortalin. RMSD of Mortaparib during the course of simulation in reference to the docked poses with Mortalin (A). Site of interaction of Mortaparib with Mortalin at different time instances during the MD simulation. It did not show any stable or significant binding.

Additional file 4: Supplementary Figure 4 (S4). Molecular docking showing interactions of Mortaparib and PARP1. Mortaparib docked to PARP1 at the cavity lined by the catalytically active residues of PARP1 and was similar to the binding of Rucaparib, Niraparib and Olaparib. PARP1 was docked with its known inhibitors Rucaparib, Niraparib and Olaparib. Mortaparib mimicked the binding behavior of other known PARP1 inhibitors.

Additional file 5: Supplementary Figure 5 (S5). Molecular docking showing interactions of PARP1 and Mortalin is shown (A) ; interacting residues of the two proteins are listed in the table on the right (B). Coimmunoprecipitation of PARP1 and Mortalin in MCF7 showing inputs of the two proteins (a), presence of mortalin in PARP1 immunocomplexes (b) and vica versa (c). The quantitative data represents mean ± SD obtained from at least three independent experiments; P-values were calculated using Student's t-test. *< 0.05, **< 0.01, and ***< 0.001 represent significant, very significant, and very very significant, respectively.

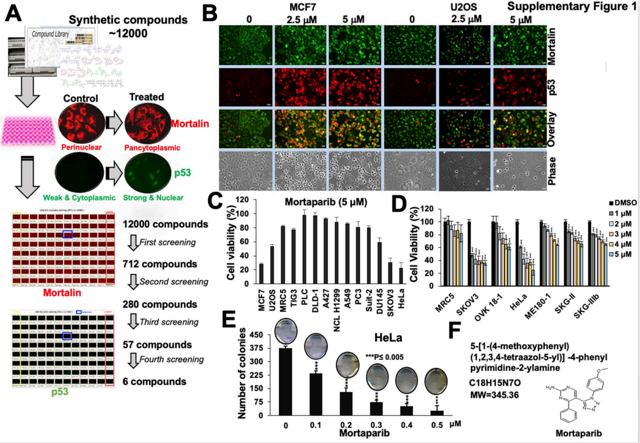

Supplementary Figure 1
A
Synthetic compounds ~12000
Compound Library
Control
Treated
Mortalin
Perinuclear
Pancytoplasmic
p53
Weak & Cytoplasmic
Strong & Nuclear
12000 compounds
First screening
712 compounds
Second screening
280 compounds
Third screening
57 compounds
Fourth screening
6 compounds
Mortalin
p53
B
MCF7
U2OS
0
2.5 μM
5 μM
Mortalin
p53
Overlay
Phase
C
Mortaparib (5 μM)
Cell viability (%)
MCF7
U2OS
MRC5
TIG3
PLC
DLD-1
A427
NCL H1299
A549
PC3
Suit-2
DU145
SKOV3
HeLa
D
Cell Viability (%)
DMSO
1 μM
2 μM
3 μM
4 μM
5 μM
MRC5
SKOV3
OVK 18-1
HeLa
ME180-1
SKG-II
SKG-IIIb
E
HeLa
***P≤ 0.005
Number of colonies
Mortaparib
μM
F
5-[1-(4-methoxyphenyl) (1,2,3,4-tetraazol-5-yl)] -4-phenyl pyrimidine-2-ylamine
C18H15N7O
MW=345.36
Mortaparib

A

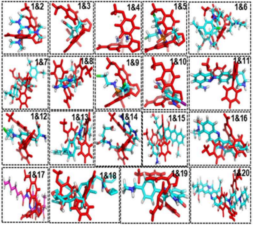

Sample-1	Mortaparib
Sample-2	Altretamine hexalen
Sample-3	Carboplatin
Sample-4	Cisplatin
Sample-5	Cyclophosphamide Cytoxan
Sample-6	Lorvotuzumab Metansine
Sample-7	Etoposide
Sample-8	Gemcitabine gemzar
Sample-9	Ifosfamide ifex
Sample-10	Inaparib
Sample-11	Niraparib
Sample-12	Melphalan
Sample-13	Olaparib
Sample-14	Pemetrexed Alimta
Sample-15	Doxorubicin doxil
Sample-16	Topotecan
Sample-17	Capecitabine xeloda
Sample-18	Docetaxel
Sample-19	Rucaparib
Sample-20	Irinotecan

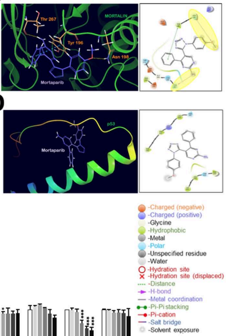

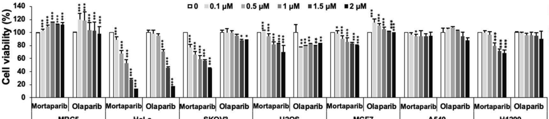

Supplementary Figure 2

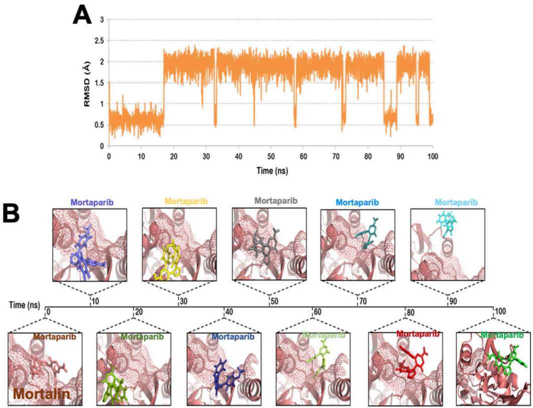

Supplementary Figure 3

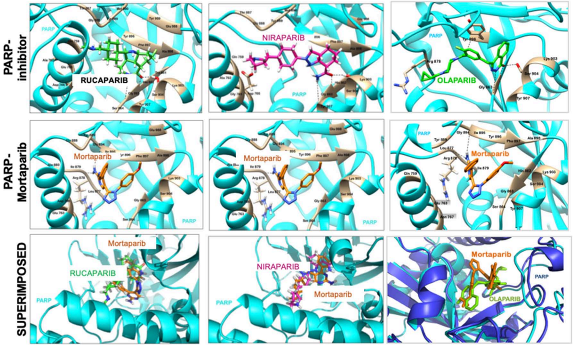

Supplementary Figure 4

A

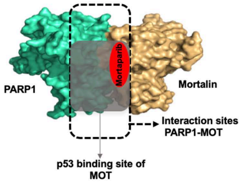

B

Mortaparib-PARP1	PARP1-Mortalin		p53 binding site to Mortalin (260-288)	Mortaparib-Mortalin
	PARP1	Mortalin		
Mortaparib binds to PARP1 C terminus(662-1014)	Glu 71	**Lys 42**		Gly 269
	Gly 72	**Arg 43**		
	Lys 73	**Ser 50**		
	Gln 74	**Ser 53**		
	Glu 79	**Glu 54**		
	Ala 81	**Gln 57**		
	Glu 82	Gln 185		
	Gly 83	Arg 186		
	Ala 84	Pro 189		
	Arg 85	Phe 190		
	Met 103	Gln 214		
	Arg 107	Leu 216		
	Asn 180	Ile 218		
	Tyr 181	Pro 220		
	Leu 182	Pro 221		
	Gly 183	Glu 222		
	Asn142	Ser 278	Ser 278	
	Ala 229	Lys 279	Lys 279	
	Lys 234	Leu 280	Leu 280	
	Ser 408	Lys 282	Lys 282	
	Lys 409	His 285	His 285	
	Ala 410	Tyr 331		
	Val 411	Asp 332		
	Ile 419	Ile 333		
		Ala 334		

C

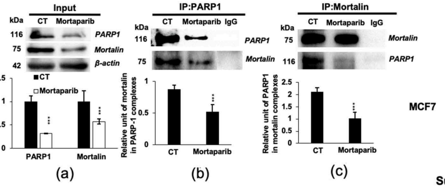

Supplementary Figure 5
